# Reliability, validity, and diagnostic accuracy of the apathy evaluation scale in chronic stroke survivors

**DOI:** 10.1186/s12888-025-06626-5

**Published:** 2025-03-05

**Authors:** Akram Jamali, Tourandokht Baluchnejadmojarad, Seyede Zohreh Jazaeri, Shiva Abedi, Hajar Mehdizadeh, Parvaneh Taghavi Azar Sharabiani, Ghorban Taghizadeh

**Affiliations:** 1 Department of Neurosciences, Faculty of Advanced Technologies in Medicine, Iran University of Medical Sciences, Tehran, Iran; 2 Cellular and Molecular Research Center, Iran University of Medical Sciences, Tehran, Iran; 3 Student Research Committee, Iran University of Medical Sciences, Tehran, Iran; 4 Brain and Cognition Clinic, Institute for Cognitive Sciences Studies, Tehran, Iran; 5 Department of Physiology, School of Medicine, Iran University of Medical Sciences, Tehran, Iran; 6 Department of Occupational Therapy, University of Social Welfare and Rehabilitation Sciences, Tehran, Iran; 7 Independent researcher, Shiraz, Iran; 8 Master of Sciences in Occupational Therapy, Shafa Yahyaeian Hospital, Iran University of Medical Sciences, Tehran, Iran; 9 Geriatric Mental Health Research Center, Department of Occupational Therapy, School of Rehabilitation Sciences, Iran University of Medical Sciences, Tehran, Iran; 10 Rehabilitation Research Center, Department of Occupational Therapy, School of Rehabilitation Sciences, Iran University of Medical Sciences (IUMS), Tehran, Iran

**Keywords:** Apathy, Stroke, AES, Psychometric properties

## Abstract

**Background:**

We aimed to determine the psychometric properties of the Apathy Evaluation Scale (AES) in chronic stroke survivors.

**Methods:**

In this study, 112 non-cognitive impairment stroke survivors participated. Acceptability, inter-rater, and test-retest reliability of the three Persian versions of AES (clinician, informant, and self-rated) were evaluated. The correlation of three AES versions with the Hospital Anxiety and Depression Scale (HADS), Modified Rankin Scale (mRS), and Barthel Index (BI) was evaluated. To assess the diagnostic accuracy of the three AES versions, stroke survivors were classified as apathetic (*n* = 43) and non-apathetic (*n* = 69) groups using the ‘diagnostic criteria of apathy’.

**Results:**

The floor and ceiling effect, skewness, and kurtosis were within acceptable range for three AES versions. Internal consistency (α = 0.88–0.91) and test-retest and inter-rater reliability (ICC_2,1_ >0.90) were acceptable for all AES versions. Standard Error of Measurement and Minimal Detectable Change values for test-retest and/or inter-rater reliability ranged 1.6–2.5 and 4.42–6.93 for three versions of AES, respectively. Significant moderate to high correlation (r or ƿ = -0.34–0.69) was found between three AES versions and HADS-D, HADS-A, BI, and mRS. The cut-off point > 34 (sensitivity = 87.5%, specificity = 72.22%, and AUC = 0.80) was derived for discriminating apathetic from non-apathetic stroke survivors based on the AES- self-rated total score.

**Conclusion:**

All three AES versions are reliable and valid screening tools to evaluate and characterize apathy in stroke survivors. The AES-self-rated had good discriminative validity for discriminating apathetic from non-apathetic subjects in non-cognitive impairment stroke survivors.

## Introduction

Stroke is one of the main causes of adult disability [1, 2]. In addition to sensory-motor impairments, stroke survivors often experience neuropsychiatric disturbances. Post-stroke apathy (PSA) is one of the most prevalent neuropsychiatric symptoms [3, 4] that are marked by deficits in behavioral, cognitive, and emotional aspects of goal-directed behavior [5]. Apathy can negatively impact functional recovery, cognitive function, and daily activities, and place a substantial burden on caregivers [4, 6–9].

Utilizing a validated scale for apathy assessment can enhance our comprehension of apathy’s clinical impact on stroke survivors and assist in refining treatment approaches. The Apathy Evaluation Scale (AES) seems to be a promising tool for clinically evaluating PSA [10]. This scale is based on a definition of apathy as a neuropsychiatric syndrome of motivation loss. What distinguishes AES from other apathy assessment tools is its provision of three different versions (AES-clinician (AES-C), AES-informant (AES-I), and AES-self (AES-S)). This feature enables the collection of data from multiple sources, leading to a more comprehensive understanding of apathy. Additionally, all AES versions are brief and provide a quantitative assessment of overall motivation loss [10]. This scale demonstrated acceptable psychometric properties in various diseases [6–8, 10–22] and is widely used in stroke studies [4, 22, 23], that make AES suitable for evaluating changes in apathy over time and assessing responses to specific treatments. Furthermore, this scale was translated into different languages (Portuguese, Japanese, German, Italian, Taiwanese, and Turkish [15, 16, 24–27]). Translation of the questionnaires is crucial for comparing cross-cultural and cross-linguistic research outcomes.

Two studies, by Marin et al. [10] and Sagen et al. [22], have reported the psychometric properties of AES in stroke survivors. Marin et al. [10] reported acceptable internal consistency, test-retest, and inter-rater reliability for all AES versions in four different groups with different clinical features, including Alzheimer’s disease (AD), stroke, depression, and elderly individuals. They also reported a moderate to strong correlation between the three AES versions, as well as a poor to moderate correlation of apathy with depression and anxiety. Sagen et al. [22] reported a Cronbach’s alpha of 0.88 for the AES-S in stroke survivors. These studies had some limitations. Marin et al. reported psychometric properties for a mixed sample and not specifically for stroke survivors. The etiology of apathy can vary depending on the underlying disease and this may affect the psychometric properties of apathy scales such as AES. For instance, PSA is associated with frontal lobe and basal ganglia lesions [28–30], whereas apathy in AD and Parkinson’s disease (PD) patients is linked to amygdala, mesolimbic dopaminergic and serotonergic lesions [31, 32]. Also, Sagen et al. reported only one aspect of psychometric properties (internal consistency), specifically for the AES-S version. Neither of these studies specifically investigated the psychometric properties, especially diagnostic accuracy of the AES in stroke survivors, nor did they determine which AES version is more suitable for measuring PSA. Therefore, our objective was to assess the acceptability, reliability, validity, and diagnostic accuracy of three versions of Persian AES in Iranian chronic stroke survivors.

## Methods

### Participants

A sample of 112 first-time stroke survivors, diagnosed through magnetic resonance imaging and neurological examination by a neurologist, was recruited from rehabilitation centers in Tehran, Iran, over a 7-month period. Inclusion criteria were: (a) ability to read and write in Persian; (b) at least 6 months post-stroke; and (c) no cognitive impairment (Montreal Cognitive Assessment (MOCA) ≥ 24) [33]. Patients with psychiatric illness, neurologic diseases other than stroke, alcohol or drug abuse, and aphasia were excluded. All participants provided written consent at the study’s outset. Ethical approval was granted by the Ethics Committee of the Iran University of Medical Sciences (Ethical Code: IR.IUMS.REC.1401.970).

### Translation

Initially, we obtained permission from Robert S. Marin, the developer of AES [10], to translate the questionnaires. Two bilingual translators, native Persian speakers, independently conducted forward translation from English to Persian. The group of translators and stroke experts discussed discrepancies and refined the two Persian AES versions collaboratively. This refinement process resulted in a synthesized version of the Persian AES that was agreed upon. Subsequently, two other native English translators fluent in Persian performed back translation from Persian to English. The back translation was reviewed again by the translators and stroke experts. To ensure the accuracy of meanings and comprehension of all questions by individuals, the prefinal version of the AES was pilot-tested on ten stroke survivors. Feedback from both patients and translators was discussed by the study panel, resulting in the issuance of the final version of the AES.

### Procedures

Data related to demographic characteristics and neuropsychological assessments were collected by an examiner. We used the MoCA [34], Hospital Anxiety and Depression Scale (HADS-D and HADS-A) [35], modified Rankin Scale (mRS) [36], and Barthel Index (BI) [37] to assess cognition, depression and anxiety, disability, and activities of daily living (ADL), respectively. The diagnosis of apathy and subject classification relied on the ‘diagnostic criteria of apathy’ (DCA) [4, 38]. The AES-S and AES-I were completed by patients and their caregivers, respectively. The AES-C was administered through face-to-face interview by two trained examiners, separately. All assessments took place in the same quiet room, with the tests administered in a random order for each subject. Adequate rest intervals (3–5 min) were provided between and within the assessments.

To assess test-retest reliability, the three AES versions were completed in two sessions with the interval of two weeks [5]. A two-week interval was chosen to minimize the likelihood of participants modifying their responses or recalling previous answers [39]. Only participants who confirmed that their symptoms had remained unchanged compared to the first test session were included in the reliability study. For the inter-rater reliability of AES-C, two examiners independently assessed participants in the first session.

We evaluated convergent validity by assessing the correlation between the three AES versions. To assess the discriminant validity, we selected HADS-D, HADS-A, BI, and mRS. Although Apathy, depression, and anxiety have overlapping symptoms, they have different constructs in nature [4]; however, previous studies have shown a significant correlation between apathy, depression, anxiety, ADL, and disability in stroke survivors [10, 15, 16, 40, 41].

To assess the diagnostic accuracy of AES for detecting apathy, stroke survivors were categorized into two groups with and without apathy based on the DCA [4, 38].

### Instruments

The AES is an 18-item questionnaire designed to measure apathy. The total score ranges from 18 to 72, with higher scores indicating more severe apathy. AES has three versions, including paper-and-pencil self-rated, paper-and-pencil informant-rated, and clinician-administered versions [10].

The HADS is a self-assessment scale for measuring anxiety and depression [42]. This scale has acceptable reliability and validity in stroke survivors [43] and Iranian breast cancer patients [35].

The mRS is a reliable single-item measure for assessing the level of global disability based on the level of disability, mobility, and dependency in ADL following stroke [36, 44].

The BI has been designed to assess an individual’s capacity to carry out ADL. This has acceptable reliability and validity in the Iranian stroke population [45].

### Statistical analysis

The minimum sample size for assessing validity was determined using an item-to-respondent ratio of 1:5 [46]. Since the AES is comprised of 18 items, a minimum sample of 90 subjects was required. The sample size needed for test-retest reliability was determined using the functional approximation method [47]. Based on a predicted reliability coefficient of 0.90 for three AES versions yielded from a pilot study, a minimum acceptable coefficient of 0.70, a power of 80%, a significance level of 0.05, and having two testing sessions, the minimum sample size of 37 subjects was needed.

The Kolmogorov–Smirnov test was used to evaluate the normal distribution of the data. Demographic and clinical characteristics were summarized using descriptive statistics. Differences between the apathetic and non-apathetic groups were analyzed using independent sample t-tests for quantitative data and chi-squared tests for qualitative data. The yielded acceptability based on floor and ceiling effects was < 15% [48] and the values of distribution skewness and kurtosis fell between − 2 and + 2 [49].

Internal consistency was determined through Cronbach’s alpha coefficient. Test-retest and inter-rater reliability for the total score of the AES were assessed using the intra-class correlation coefficient (ICC_2,1_) with a 95% confidence interval (CI_95%_). The minimal acceptable value for Cronbach’s alpha and ICC was set at 0.70 [50]. Test-retest and inter-rater reliability for each item of the AES was assessed using the kappa coefficient, with values categorized as moderate (0.41–0.60), substantial (0.61–0.80), and perfect (0.81–1.00) [51]. To determine test-retest and inter-rater reliability, the accuracy of the AES scores was derived using the standard error of measurement (SEM = SD pooled √1– ICC) [52] and minimal detectable change (MDC_95%_ = SEM×√2 × 1.96) [53] SEM value < 1/2 SD was considered as an acceptable precision [50]. The Pearson correlation coefficient was used to assess the correlation between all versions of the AES, HADS-D, HADS-A, and BI, while the Spearman correlation coefficient was used to investigate the correlation between all versions of the AES and the mRS. A correlation coefficient of 0.3–0.5 indicates a moderate correlation, whereas a coefficient greater than 0.5 indicates a strong correlation [54].

To evaluate the agreement between the three AES versions and the DCA in measuring apathy, we calculated frequencies and percentage frequencies. Diagnostic accuracy was evaluated using Receiver Operating Characteristic curve analysis and Area Under the Curve (AUC) value calculation. An AUC value > 0.70 was considered acceptable [55]. This analysis assessed the ability of AES to differentiate between apathetic and non-apathetic stroke survivors based on the DCA. This analysis also provided sensitivity, specificity, positive predictive value (PPV), and negative predictive value (NPV). Our significance level was set at *p* < 0.05.

## Results

In Tables 1 and 2, the demographic and stroke-related characteristics of stroke survivors were shown. Forty-three patients (38%) had apathy based on the ‘apathy diagnostic criteria’ that demonstrated higher levels of disability, depression, and anxiety and a decreased level of independence in ADL.

**Table 1:** Demographic characteristics of study participants in each group

Variable		Total (*n* = 112)	Non-apathetic group (*n* = 69)	Apathetic group (*n* = 43)	*P*_value
Age (year), mean ± SD		56.88 ± 11.7	55.62 ± 10.57	58.89 ± 13.18	0.15
Sex, *n* (%)	female	44 (39.29)	28 (40.78)	16 (37.21)	0.72
	male	68 (60.71)	41 (59.42)	27 (62.79)	
Educational level, *n* (%)	university	33 (29.46)	22 (31.88)	11 (25.58)	0.48
	no university	79 (70.54)	47 (68.12)	32 (74.42)	
Employment status, *n* (%)	employed	40 (35.71)	29 (42.03)	11 (25.58)	0.08
	unemployed	72 (64.29)	40 (57.97)	32 (74.42)	
Marital status, *n* (%)	single	16 (14.29)	10 (14.49)	6 (13.95)	0.94
	married	96 (85.71)	59 (85.51)	37 (86.05)	

**Table 2:** Clinical characteristics of study participants in each group

Variable		Total (*n* = 112)	Non-apathetic group (*n* = 69)	Apathetic group (*n* = 43)	P_value
Affected side, *n* (%)	right	66 (58.93)	40 (57.97)	17 (39.53)	0.06
	left	46 (41.07)	29 (42.03)	26 (60.47)	
Time since stroke onset (month), *n* (range)		75.07 ± 60.02 (6-312)	79.48 ± 64.40 (6-312)	68 ± 52.18 (6-216)	0.34
Lesion type, *n* (%)	ischemic	75 (66.96)	48 (69.57)	27 (62.79)	0.93
	hemorrhage	37 (33.04)	24 (34.78)	13 (30.23)	
MRS, *n* (%)	Score 0	11 (9.82)	10 (14.49)	1 (2.33)	**0.00**
	Score 1	55 (49.11)	40 (57.97)	15 (34.88)	
	Score 2	25 (22.32)	13 (18.84)	12 (27.91)	
	Score 3	15 (13.39)	5 (7.25)	10 (23.26)	
	Score 4	5 (4.46)	1 (1.45)	4 (9.30)	
	Score 5	1 (0.89)	0 (0)	1 (2.33)	
BI, mean ± SD		91.61 ± 16.33	95.65 ± 10.36	85.12 ± 21.48	**0.001**
HADS-D, mean ± SD		5.5 ± 4.45	4.06 ± 3.59	7.81 ± 4.74	**0.00**
HADS-A, mean ± SD		5.15 ± 4.17	3.71 ± 3.44	7.46 ± 4.24	**0.00**
AES-C, mean ± SD	Test	39.12 ± 11.24	33.36 ± 7.89	48.37 ± 9.55	**0.00**
	Re-test	38.81 ± 11.16	33.4 ± 8.58	47.49 ± 9.21	**0.00**
	Second examiner	38.39 ± 10.2	33.05 ± 7.23	46.95 ± 8.31	**0.00**
AES-S, mean ± SD	Test	35.82 ± 11.44	30.11 ± 7.47	44.97 ± 10.8	**0.00**
	Re-test	35.75 ± 11.74	29.55 ± 7.4	45.72 ± 10.52	**0.00**
AES-I, mean ± SD	Test	38.90 ± 11.16	33.67 ± 8.04	47.28 ± 10.37	**0.00**
	Re-test	38.42 ± 11.44	33.07 ± 8.20	47.02 ± 10.69	**0.00**

MRS: modified rankin scale, BI: Barthel index, HADS-D: Depression subscale of Hospital Anxiety and Depression Scale, HADS-A: Anxiety subscale of Hospital Anxiety and Depression Scale, AES-C: Apathy Evaluation Scale- Clinician version, AES-S: Apathy Evaluation Scale- Self-rated, AES-I: Apathy Evaluation Scale- Informed version

The floor effect was 0%, 3.57%, and 2.68% for AES-C, AES-S, and AES-I, respectively while the ceiling effect was 0.89% for the three AES versions. The skewness values were in acceptable range (AES-C = 0.63, AES-S = 0.85, and AES-I = 0.58). The kurtosis values were − 0.05, 0.48, and − 0.02 for AES-C, AES-S, and AES-I, respectively.

The Cronbach’s alpha coefficient was 0.88, 0.90, and 0.91 for AES-C, AES-S, and AES-I, respectively. The ICC_2,1_ values for the test-retest and inter-rater reliability of the AES-C were 0.95 (CI95% =0.92–0.96) and 0.97 (CI95% =0.96–0.98), respectively. The ICC_2,1_ values for the test-retest reliability of the AES-S and AES-I were 0.97 (CI95% =0.95–0.98) and 0.98 (CI95% =0.96–0.98), respectively. Kappa agreement coefficients for all items of the three AES versions in test-retest and/or inter-rater were ranged from 0.41 to 0.89. The SEM values for test-retest and inter-rater of AES-C were 2.5 and 1.86, respectively. The SEM values for the test-retest of AES-S and AES-I were 2 and 1.6, respectively. The MDC values for the test-retest and inter-rater reliability of AES-C, and the test-retest reliability of AES-S and AES-I, were 6.93, 5.15, 5.55, and 4.42, respectively.

There were strong significant inter-correlations between the three AES versions (*r* = 0.75–0.85) as well as moderate to strong significant correlation between AES with HADS-D, HADS-A, MRS, and BI (r or ƿ =-0.34 to *r* = 0.69) (Table 3).

**Table 3:** Construct validity of the three versions of the Persian apathy evaluation scale (AES) (*N* = 112)

	AES-C *r* or ƿ	AES-S *r* or ƿ	AES-I *r* or ƿ
AES-C	-	-	-
AES-S	0.85**	-	-
AES-I	0.75**	0.81**	-
MRS	0.54**	0.53**	0.43**
BI	-0.48**	-0.48**	-0.34**
HADS-D	0.65**	0.68**	0. 69**
HADS-A	0.59**	0.63**	0.61**

AES-C: Apathy Evaluation Scale- Clinician version, AES-S: Apathy Evaluation Scale- Self-rated, AES-I: Apathy Evaluation Scale- Informed version, MRS: modified Rankin Scale, BI: Barthel index, HADS-D: Depression subscale of Hospital Anxiety and Depression Scale, HADS-A: Anxiety subscale of Hospital Anxiety and Depression Scale

**P < 0.001

The agreement percentages between AES-C, AES-S, and AES-I with the DCA in subjects with apathy were 90.7%, 88.4%, and 90.7%, respectively (Table 4).

**Table 4:** The degree of agreement between three versions of the Persian apathy evaluation scale (AES) with diagnostic criteria of apathy in identifying apathy (*N* = 112)

Scale		Diagnostic criteria of apathy		
		With apathy (*N* = 43)	Without apathy (*N* = 69)	Total (*N* = 112)
AES- clinician version	With apathy	39 (90.7%)	24 (34.8%)	63 (56.25%)
	Without apathy	4 (9.3%)	45 (65.2%)	49 (43.75%)
AES-Self version	With apathy	38 (88.4%)	17 (24.6%)	55 (49.11%)
	Without apathy	5 (11.6%)	52 (75.4%)	57 (50.89%)
AES-Informed version	With apathy	39 (90.7%)	32 (46.4%)	71 (63.39%)
	Without apathy	4 (9.3%)	37 (53.6%)	41 (36.61%)

The AES-S had acceptable sensitivity (87.5%), specificity (72.22%), and AUC (0.80, *p* < 0.001) to classify chronic stroke survivors with and without apathy (Table 5).

**Table 5:** Sensitivity and specificity of three versions of the Persian apathy evaluation scale (AES) (*N* = 112)

	AES-Clinician version	AES-Self version	AES-Informed version
Sensitivity (95%CI)	90 (76.3–97.2)	87.5 (73.2–95.8)	90 (76.3– 97.2)
Specificity (95%CI)	62.5 (50.3–73.6)	72.22 (60.4–82.1)	51.39 (39.3–63.3)
Positive predictive value (95%CI)	21.1 (16.3–26.8)	25.9 (19.2–34.1)	17.1 (13.7–21)
Negative predictive value (95%CI)	98.3 (95.6–99.3)	98.1 (95.8–99.2)	97.9 (94.7–99.2)
Positive likelihood ratio (95%CI)	2. 40 (1.75–3.29)	3.15 (2.13–4.65)	1.85 (1.43–2.40)
Negative likelihood ratio (95%CI)	0.16 (0.06–0.41)	0.17 (0.07–0.4)	0.19 (0.07–0.51)
Area Under the Curve (AUC) (P-value)	0.76 (< 0.001)	0.80 (< 0.001)	0.71 (< 0.001)

cut-off of AES: 34

## Discussion

Given the high prevalence (up to 70%) of apathy [23], and its significant impact on various aspects of functional activities, there is a critical need for standardized tools to measure apathy for early identification and routine clinical use. AES is one of the most frequently used tools to assess apathy in stroke survivors [4] and has demonstrated acceptable validity and reliability in various diseases and in different languages. To the best of the authors’ knowledge, the psychometric properties of AES have not been comprehensively investigated in stroke survivors. The results of this study indicated that all three AES versions have high acceptability, acceptable internal consistency, and test-retest and/or inter-rater reliability. Additionally, all AES versions showed moderate to strong correlations with depression, anxiety, disability, and ADL. Only AES-S has acceptable AUC, sensitivity and specificity to differentiate stroke survivors with and without apathy.

The three AES versions demonstrated an acceptable range of skewness and kurtosis that implied an acceptable level of asymmetry and ‘peaked-ness’ in the distribution, the absence of significant errors in measuring apathy, and an accurate representation of true results. There was no marked floor or ceiling effect; therefore, all AES versions can distinguish between severe and mild cases of apathy and can detect changes in apathy over time or in response to interventions. These results were consistent with the previous studies conducted on multiple sclerosis and PD patients using the AES-S [6, 18].

We observed acceptable internal consistency in all AES versions that exhibit homogeneity and coherence in all items in the concept of apathy. This finding is consistent with the reported internal consistency by Marin et al. for the three AES versions in a mixed population [10] and by Sagen et al. for AES-S among stroke survivors [22].

This study exhibited acceptable test-retest and/or inter-rater reliability and kappa agreement coefficients for the total score and each item of all AES versions. In the current study, a two-week interval was employed for test-retest reliability, which is the most frequently recommended time interval in previous studies [5, 21]. A very short time interval for test-retest increases the likelihood of recall bias due to the influence of memory, whereas a longer interval enhances the potential for clinical change in patients [39]. Our results showed that within this appropriate time interval, all versions exhibited acceptable stability and were not significantly affected by the genuine and learning effects. These findings closely resemble the results of Marin et al.‘s study in a mixed population, and other studies have been done on AD and psychosis patients for AES-C and on myotonic dystrophy patients for AES-I and AES-S [10, 14, 16, 21].

The SEM represents the variability in the measurement that can be attributed to measurement error. The results of the SEM analysis demonstrated low variability and adequate precision in participant responses during the test-retest and inter-rater assessments which is consistent with the previous studies on PD and myotonic dystrophy diseases [12, 21]. The MDC values ranged from 5.43 to 6.63 for all versions. These values represent the minimum needed score change to confirm that the observed change is a result of real change rather than instrument error.

In agreement with Marin et al.‘s study, strong inter-correlations were observed among three AES versions indicating that all versions of AES, despite their different sources of testing, measure the same concept [10]. Consistent with previous studies [10, 15, 16], a strong positive correlation was observed between apathy and depression. Apathy and depression are two distinct concepts with different anatomical and neurobiological mechanisms but often related psychological constructs such as loss of pleasure and energy. The overlap between depression and apathy can develop during the chronic phase of stroke, and it’s noteworthy that our study subjects were in the chronic phase. Results revealed a strong positive correlation between apathy and anxiety in stroke survivors, which is consistent with previous studies [4, 10]. This result may be because apathy and anxiety are coincidentally observed in stroke survivors, and a bidirectional correlation can exist between them. High levels of anxiety can reduce motivation and interest in activities. Conversely, apathy can contribute to increased worry about their inability to participate in meaningful activities. Our results indicate a moderate-to-strong significant correlation between apathy and ADL in stroke survivors. This negative correlation between apathy and ADL aligns with findings from previous studies targeting stroke patients [40]. A reduction in motivation and interest can potentially lead to a decreased desire to engage in ADL. Additionally, we observed a moderate-to-strong significant correlation between apathy and disability resulting from stroke. More disability may be due to the extensive involvement of brain areas, potentially leading to an increased likelihood of apathy. Also, more disability can lead to increased hopelessness for recovery and increased apathy.

The acceptable agreement in diagnosing apathetic stroke survivors was observed between all versions of AES and the DCA. The AES-S, compared with the AES-C and AES-I (with unacceptable specificity), demonstrated acceptable sensitivity, specificity, and AUC values with a high NPV but a low PPV. The acceptable specificity of AES-S may be attributed to the fact that this study focused on patients with no cognitive impairment. These findings align with previous studies that have reported AES-S as a more sensitive version compared to informant and clinician versions in subjects with normal cognition [8]. Considering the combination of a high NPV and a low PPV in AES-S, this may be used clinically as a screening tool to rule out the presence of apathy when the results are less than the cutoff score. Nevertheless, in cases where the results are above the cutoff score, further assessment by clinicians’ is needed to prove apathy. Furthermore, PPV and NPV are heavily influenced by the prevalence of the disorder in the population, and low PPV in our results may be due to the low prevalence of apathy (38%) in our study. Therefore, AES-C and AES-I do not seem to provide additional diagnostic value beyond AES-S and thus may not be necessary for clinical use in subjects with normal cognitive function.

The limitations of this study include the highly selective nature of the participants, as it did not include stroke survivors with cognitive impairments, low levels of independence, or language difficulties such as aphasia. This underscores the need for future studies to include stroke survivors with more significant limitations.

## Conclusion

In summary, the results of the present study demonstrated that the three AES versions have acceptable reliability and validity for stroke survivors. While only AES-S has acceptable sensitivity, specificity, and AUC to discriminate stroke survivors with and without apathy.

## Data Availability

No datasets were generated or analysed during the current study.

## Abbreviations

AES: Apathy Evaluation Scale
AES-C: Apathy Evaluation Scale- clinician administered version
AES-I: Apathy Evaluation Scale- informant rated version
AES-S: Apathy Evaluation Scale- self rated version
PSA: Post-stroke apathy
MOCA: Montreal Cognitive Assessment
HADS-D: Hospital Anxiety and Depression Scale-Depression
HADS-A: Hospital Anxiety and Depression Scale-Anxiety
mRS: modified Rankin Scale
BI: Barthel Index
ICC2,1: intra-class correlation coefficient
AD: Alzheimer’s disease
PD: Parkinson’s disease
ADL: Activities of Daily Living
DCA: diagnostic criteria for apathy
CI95%: confidence interval
SEM: standard error of measurement
MDC: minimal detectable change
AUC: Area Under the Curve
PPV: positive predictive value
NPV: negative predictive value
